# Complete regression of renal tumour following ligation of an accessory renal artery during repair of an abdominal aortic aneurysm

**DOI:** 10.1308/003588412X13373405384972

**Published:** 2012-09

**Authors:** J Sammut, E Ahiaku, DT Williams

**Affiliations:** ^1^Betsi Cadwaladr University Health Board,UK; ^2^School of Medical Sciences, Bangor University, Wales,UK

**Keywords:** Renal cell carcinoma, Abdominal aortic aneurysm, Accessory renal artery

## Abstract

The existence of concomitant intra-abdominal pathology with abdominal aortic aneurysms is not uncommon. The optimal management is often controversial. We describe the successful treatment of a case of an abdominal aortic aneurysm (AAA) associated with a renal tumour without performing a nephrectomy. An accessory lower pole renal artery supplying the tumour was ligated at the time of open AAA repair. The lower pole renal tumour (suspected renal cell carcinoma) reduced in size dramatically and progressively on follow-up computed tomography and the patient remains well at over two years after surgery. The successful treatment of the two conditions in such a manner represents an alternative management strategy and adds to the options available in selected patients who present with challenging and unusual pathology.

An abdominal aortic aneurysm (AAA) is sometimes discovered incidentally when investigations are performed for other reasons, most commonly for known or suspected intra-abdominal pathology. Alternatively, other intra-abdominal pathology may be discovered incidentally during the diagnostic workup for the AAA. In any case, the clinician is faced with a dilemma when two life threatening conditions are discovered together: how best to treat the patient. A common scenario is an intra-abdominal malignancy in association with an AAA. When both conditions require operative intervention, the order in which to do so is dictated by which condition poses the greatest short-term risk to life although this may not always be clear. Repair of the AAA often takes priority as the risk of rupture in the intervening period is considerable.^1^

Simultaneous treatment of both conditions is also an option. However, when considering bowel resections, this is generally considered unattractive, given the enhanced risk of graft contamination and subsequent infection from bowel pathogens at the time of surgery. On the other hand, the upper urinary tract is usually sterile. A simultaneous nephrectomy at the time of AAA repair is therefore more attractive. This has been described by several authors and is an accepted treatment option for patients diagnosed with a renal tumour and AAA.^2,3^ Some advocate simultaneous treatment whenever possible when dealing with renal tumours in association with AAA although clear guidance on optimal management does not exist.^2^ Nephron sparing or partial nephrectomy is also an option in selected patients.^4^

We describe a case of AAA repair when ligation of an accessory renal artery at the time of surgery resulted in the resolution of a lower pole renal tumour. We believe this is the first published report describing the successful treatment of the two conditions in such a manner.

## Case history

**Figure 1:**
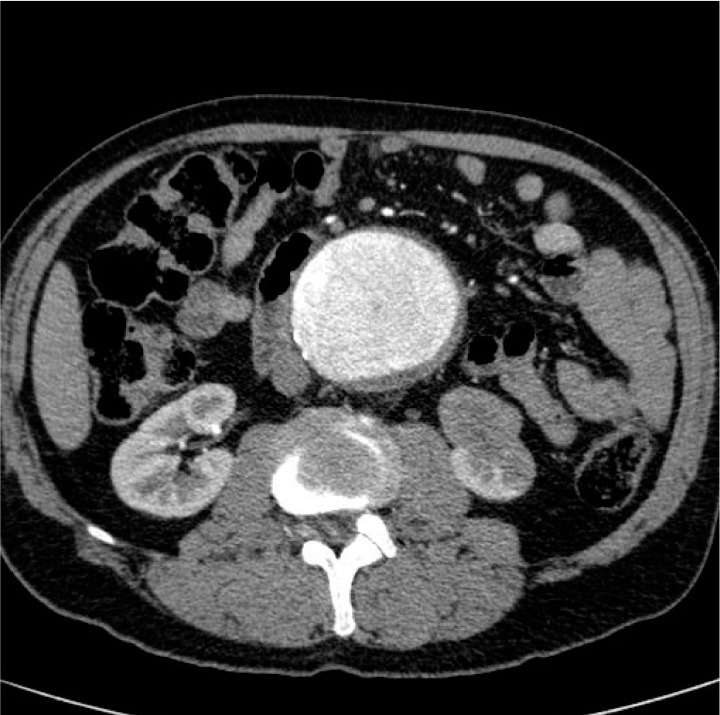
Computed tomography of abdomen demonstrating left-sided lower pole renal tumour (highly suspicious of renal cell carcinoma) together with large abdominal aortic aneurysm

A 63-year-old man was referred for vascular surgical assessment following discovery of an AAA on ultrasonography of the abdomen. The ultrasonography had been requested as an investigation for abdominal symptoms. The 7.4cm AAA was an incidental finding. The patient had a history of chronic lower back pain (having had surgery for spondylolisthesis many years previously) and hypertension. More recently, he had been diagnosed with inflammatory bowel disease. Computed tomography (CT) was requested to define the aneurysm more accurately prior to surgical intervention. This revealed an 8.2cm infrarenal AAA with a 2cm long conical neck and an angle of 50º in the sagittal plane. There was an incidental finding of a 3cm soft tissue mass involving the lower pole of the left kidney, highly suspicious of a renal cell carcinoma (Fig 1).

**Figure 2:**
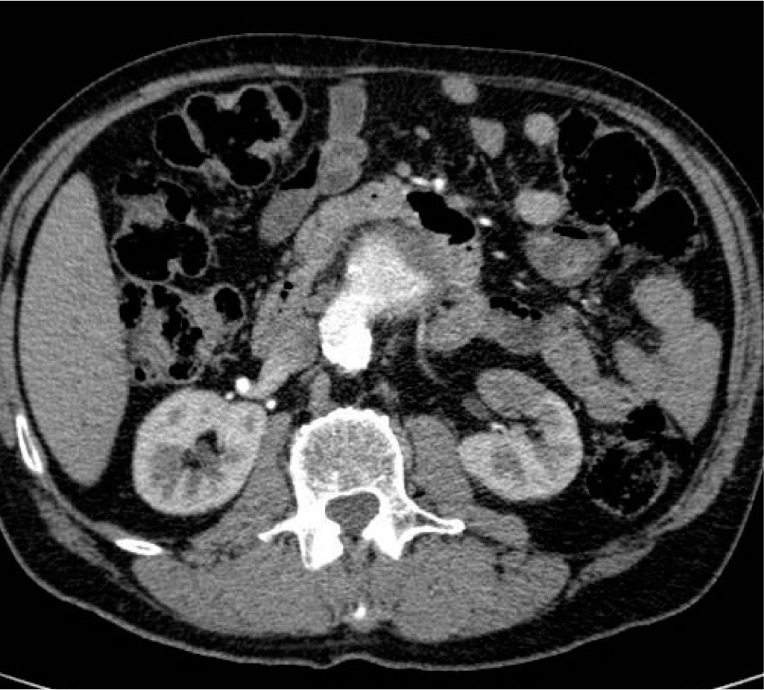
More proximal computed tomography demonstrating left accessory renal artery arising from origin of abdominal aortic aneurysm that was supplying the lower pole tumour, the superior aspect of which can also be visualised

**Figure 3:**
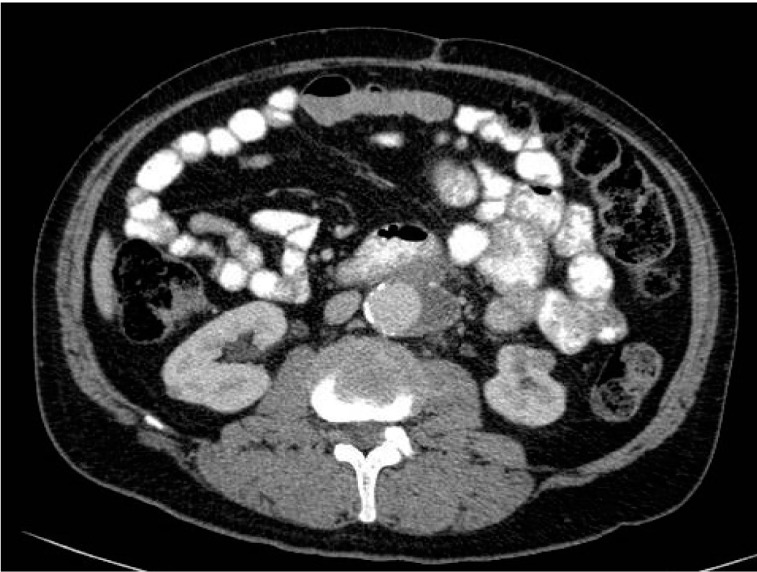
Computed tomography of renal tumour at three months after surgery with the vascular graft in situ and the lesion in lower pole of left kidney, showing cystic change and reduction in size (as a result of tumour necrosis following ligation of accessory renal artery)

**Figure 4:**
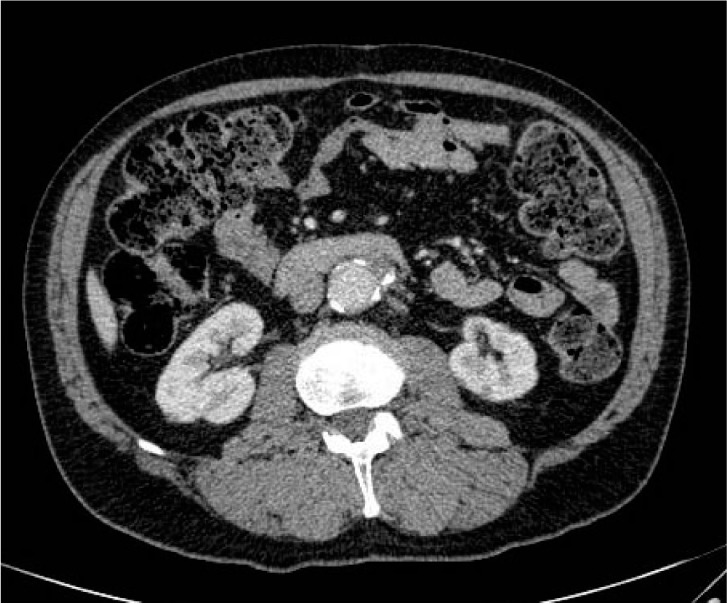
Computed tomography of tumour at 17 months after surgery: The upper aspect of the tube graft with collapsed aneurismal sac surrounding it can be seen; the left lower pole renal lesion is no longer demonstrable.

The AAA was deemed only suitable for endovascular repair employing fenestrated graft or open repair. The issues regarding treatment of the renal tumour and AAA were discussed. The patient underwent elective transperitoneal open repair three weeks later. A straight 18mm graft was used (Gelsoft™ Plus; Vascutek, Inchinnan, UK) to repair the aneurysm. A high accessory renal artery to the left lower pole was ligated during surgery. This vessel could be seen on the pre-operative CT (Fig 2). He made an uneventful post-operative recovery and was discharged on day 7. Follow-up CT revealed initial cystic change and marked reduction in renal tumour size at three months (Fig 3). There was a further decrease in the size of the residual lesion at eight months post-operatively and the lesion was no longer identifiable on the most recent CT at 17 months following surgery (Fig 4). The patient remains well at over two years after surgery.

## Discussion

Generally, where nephrectomy is considered the only potentially curative option, this should be offered at the time of AAA repair.^2^ When dealing with left-sided tumours confined radiologically to the kidney, a retroperitoneal approach for both nephrectomy and AAA repair has previously been suggested as optimal whereas a transperitoneal approach was recommended for right-sided tumours or those patients with suspected intraperitoneal tumour spread.^3^ However, with the advent of laparoscopic nephrectomies and endovascular aneurysm repair (EVAR), treatment options have widened. For example, EVAR followed by a staged laparoscopic nephrectomy during the same hospital stay was described in 2009 with a good result.^5^ Partial nephrectomy is an attractive option when anatomically possible as some kidney function will be preserved in the operated kidney.^4^ The risks and benefits of the procedure(s) and the final management will depend on the particular features of the case and discussion with the patient.

Our case is unusual in that the renal tumour was relatively small (3cm) and was confined to a lower pole that appeared to derive its blood supply from a prominent accessory renal artery that was coming off the neck of the AAA (Fig 2). The management was discussed prior to performing surgery. There appeared to be three principle management options:

1. radical nephrectomy at the time of AAA repair
2. partial nephrectomy at the time of AAA repair or
3. ligation of accessory renal artery supplying tumour at the time of AAA repair with close monitoring of the renal mass post-operatively

It was felt that the AAA posed the greatest short-term risk to the patient’s health (ie management of renal tumour should not precede AAA repair). Loss of the entire kidney (ie option 1) was not essential anatomically so it was felt that the possibility of preserving renal function should be offered. However, performing a partial nephrectomy would have added considerable time to the procedure and, given the patient’s general health, there were concerns about the risks of such a long anaesthetic. The decision was therefore made to ligate the lower pole vessel supplying the tumour during AAA repair with post-operative CT surveillance of the renal mass. If the renal tumour persisted, the patient would be offered a nephron sparing nephrectomy at a later date.

## Conclusions

To our knowledge, this is the first case describing the successful treatment of a renal tumour associated with an AAA by ligating an accessory renal artery at the time of AAA repair. Ligation of the vessel resulted in infarction of the lower pole with complete regression of the renal tumour and preservation of renal function. It also obviated the need for a nephrectomy and its associated risks. Detailed pre-operative anatomical imaging employing CT angiography can identify patients suitable for bespoke interventions when presenting with unusual pathology.
